# Programmed Necrosis: A Prominent Mechanism of Cell Death following Neonatal Brain Injury

**DOI:** 10.1155/2012/257563

**Published:** 2012-05-16

**Authors:** Raul Chavez-Valdez, Lee J. Martin, Frances J. Northington

**Affiliations:** ^1^Neonatal Research Laboratory, Department of Pediatrics, Johns Hopkins University School of Medicine, Baltimore, MD 21287-3200, USA; ^2^Division of Neonatology, Texas Tech University Health Sciences Center, Odessa, TX 79763, USA; ^3^Department of Neuroscience, Johns Hopkins University School of Medicine, Baltimore, MD 21287-3200, USA; ^4^Division of Neuropathology, Johns Hopkins University School of Medicine, Baltimore, MD 21287-3200, USA; ^5^Department of Pathology, Johns Hopkins University School of Medicine, Baltimore, MD 21287-3200, USA

## Abstract

Despite the introduction of therapeutic hypothermia, neonatal hypoxic ischemic (HI) brain injury remains a common cause of developmental disability. Development of rational adjuvant therapies to hypothermia requires understanding of the pathways of cell death and survival modulated by HI. The conceptualization of the apoptosis-necrosis “continuum” in neonatal brain injury predicts mechanistic interactions between cell death and hydrid forms of cell death such as programmed or regulated necrosis. Many of the components of the signaling pathway regulating programmed necrosis have been studied previously in models of neonatal HI. In some of these investigations, they participate as part of the apoptotic pathways demonstrating clear overlap of programmed death pathways. Receptor interacting protein (RIP)-1 is at the crossroads between types of cellular death and survival and RIP-1 kinase activity triggers formation of the necrosome (in complex with RIP-3) leading to programmed necrosis. Neuroprotection afforded by the blockade of RIP-1 kinase following neonatal HI suggests a role for programmed necrosis in the HI injury to the developing brain. Here, we briefly review the state of the knowledge about the mechanisms behind programmed necrosis in neonatal brain injury recognizing that a significant proportion of these data derive from experiments in cultured cell and some from in vivo adult animal models. There are still more questions than answers, yet the fascinating new perspectives provided by the understanding of programmed necrosis in the developing brain may lay the foundation for new therapies for neonatal HI.

## 1. Introduction

Neonatal hypoxic-ischemic encephalopathy (HIE) is a significant cause of mortality and morbidity in the pediatric population [1]. The therapeutic options for neonatal HIE are limited in part because the mechanisms of cellular degeneration in the immature brain are not fully understood. These mechanisms resulting from ischemia-reperfusion, oxidative stress, excitotoxicity and inflammation among others, activate or coactivate multiple pathways of cell death. Although, necrosis was initially described as the most prominent form of cellular degeneration following neonatal hypoxia-ischemia (HI) [2, 3], research emphasis switched to the study of apoptosis (programmed cell death type I) and autophagy largely due to advances in cell biology and to experimental animal studies on the molecular dissection of pathways for apoptotic and autophagocytic initiation and execution. The significance of necrosis in neonatal HI has been difficult to assess because of the presumed lack of a measurable regulatory pathway; however, the pathological evidence for necrosis has been well documented following HI [4, 5]. We now know that necrosis can be regulated and programmed and that many components of the regulatory pathways are shared between different types of cell death opening a new window of opportunity for examination/reexamination of the cell death mechanisms in the neonatal brain with the goal of finding novel targets for therapy. 

Based on morphological and biochemical data, we conceptualized that neurodegeneration in the neonatal brain is best classified according to an apoptosis-necrosis cell death “continuum” [6] and proposed that programmed cell necrosis (also called necroptosis in cell cultures) has a prominent contribution to neurodegeneration following HI [7]. It is certain that neonatal HI injury evolves through many cell death chreodes influenced by the dynamic injury landscape of the developing brain [8] and the mechanisms of injury in human neonatal HI are more complex than previously anticipated from experimental animal models. The accurate identification of the various cell death chreodes including programmed necrosis and their mechanisms unfolding within the immature brain will, in all likelihood, provide fresh rationale for the development of molecular-based therapies for neonatal brain injury following HI.

## 2. Programmed Cell Necrosis in Neonatal HI

Programmed necrosis as such has only recently been recognized as an important mechanism of injury in the immature brain following HI [7], however many aspects of programmed necrosis signaling have been comprehensively analyzed by the neonatal brain injury research community over the past decade (Table 1). This work piggybacks on a tremendous body of cell culture data on the mechanisms and contributions of programmed necrosis to cell death since the publication of 3 seminal papers in 1998, 2000, and 2003 [9–11]. This literature has been extensively reviewed recently [12–17].

We proposed that this novel regulated programmed necrosis, lies along the apoptosis-necrosis “continuum” and contributes meaningfully to several forms of acute neonatal brain injury [7, 18]. The death domain containing serine/threonine kinase, receptor interacting protein (RIP)-1, is central to the most well-described forms of programmed necrosis. Its kinase activity is selectively blocked by necrostatins and this affords protection against RIP-1-dependent forms of cell death [19, 20]. Blockade of RIP-1 kinase using necrostatin provides protection in adult animal models of myocardial ischemia and ischemic and traumatic brain injury [18, 21, 22]. Similarly in neonatal HI, blockade of RIP-1 kinase attenuates brain injury at delayed stages in forebrain, hippocampus, and thalamus [7]. The necrostatins have been a major tool for investigation of RIP-1-dependent cell death pathways, however there are other tools that are now being used to explore RIP-1-dependent pathways and these will be discussed below.

The specific allosteric blockade of the kinase activity of RIP-1 has been studied extensively in cell cultures to demonstrate distinct signaling pathways leading to morphologic necrosis; however, many forms of necrosis in cultured cells, appear to proceed with different kinetics and not all are RIP-1 kinase dependent [23]. Some of the known and suspected RIP-1-independent programmed necrosis pathways include (i) caspase recruitment domain (ASC)-mediated necrosis, that is dependent of the non-catalytic activity of caspase-1 [24]; (ii) p53-cathepsin Q-mediated necrosis, that is activated by reactive oxygen species (ROS) and deoxyribonucleic acid (DNA) damage [25]; (iii) apoptosis inducing factor (AIF) and poly(ADP-ribose)polymerase-1-(PARP-1-) dependent pathways (controversy exists over the role of RIP-1 in these forms of programmed necrosis) [26–30]. These pathways to necrosis will not be emphasized since RIP-1-dependent pathways are the focus of this paper and have been most extensively studied.

### 2.1. The Many Faces of RIP-1: Making the Decision between Living or Dying

**Figure 1:**
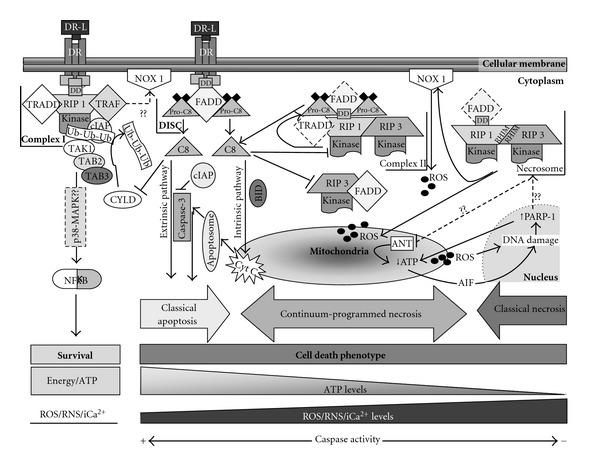
The role of RIP-1 in programmed necrosis. Following neonatal HI, members of the TNFR superfamily (also called death receptors, DR) are activated by their ligands (DR-L) (i.e., FasL, TNF-*α*). In the setting of energy sufficiency and upon TNFR activation, TNFR undergoes a conformational modification of its cytoplasmic portion allowing the interaction with receptor interacting protein (RIP)-1 with the death domain (DD), TNFR-associated death domain (TRADD), and TNFR-associated factor (TRAF)-2 and -5. They in turn recruit the cellular inhibitor of apoptosis (cIAP) forming the complex I. cIAP inhibits caspase-3 activation and allows ubiquitylation of RIP-1. Next, transforming growth factor-*β*-activated kinase (TAK)-1/TAK-1 binding protein (TAB)-2/TAB-3 form a complex that binds to ubiquitin residues on RIP-1 and activates nuclear factor-*κ*B (NF*κ*B). This may occur via a p38 mitogen-activated-protein-kinase-(p38-MAPK-) dependent pathway. Complex I may interact with NADPH oxidase (NOX 1) producing ROS, also possibly triggering programmed necrosis. Deubiquitylation of RIP-1 by the enzyme cylindromatosis (CYLD) favors the transformation of complex I to complex II binding to the internalized death-inducing-signaling-complex (DISC, formed by FAS-associated protein with death domain (FADD) and procaspase-8 (Pro-C8)) and RIP-3 (Complex II). If energy is only partially insufficient, RIP-1 activates caspase-8 (C8) signaling for classical apoptosis via intrinsic (where truncated BID binds to the outer mitochondrial membrane allowing the release of cytochrome C (Cyt C) and triggering apoptosome formation) or extrinsic pathway resulting in caspase-3 activation. In this setting, caspase-8 cleaves RIP-1 and RIP-3 preserving signal for apoptosis; however, if energy failure evolves, caspase activity declines favoring (i) preservation of the RIP-1 kinase activity, (ii) decrease in RIP-3/FADD constitutive interaction, and (iii) autophosphorylation between RIP-1 with RIP-3 at the RIP homotypic interaction motif (RHIM) forming the necrosome. Necrosome induces reactive oxygen species (ROS) production via activation of NOX 1 at the cellular membrane or direct effects in the mitochondria. ROS cause DNA alkylation increasing activation of calpain-dependent poly(ADP-ribose)polymerase-1 (PARP-1) which is normally required for DNA repair. Hyperactivation of PARP-1 induces ATP depletion and apoptosis-inducing factor (AIF) translocation from the mitochondria to the nucleus which in turn produces further DNA damage and PARP-1 activation. Necrosome formation is a potential intermediate step that follows PARP-1 activation potential intermediate steps that follow PARP-1 activation. There is some evidence that it produces ATP depletion via inhibition of adenine nucleotide translocase (ANT) in the inner mitochondrial membrane. Mitochondrial dysfunction is likely at the core of the events resulting in programmed necrosis.

Maximal execution of RIP-1-mediated activation of programmed necrosis occurs in the setting of caspase inhibition [20, 31] which can occur as a consequence of pharmacologic inhibition or significant mitochondrial dysfunction and adenosine-5′-triphosphate (ATP) depletion [32–35]. Others and we have hypothesized that energy failure interrupts the neonatal brain's proclivity to apoptosis [6, 32, 33, 36] resulting in the hybrid, “continuum” cell death, or programmed necrosis morphology, possibly via activation of RIP-1 kinase [7]. Following activation of tumor necrosis factor (TNF) receptor (TNFR), RIP-1 signaling leads to a variety of cell fates and has been, for the most part, studied in cell culture [16]. In the setting of energy sufficiency, activation of members of TNFR superfamily (i.e; TNFR1, Fas death receptor (Fas-DR)) by their cognate ligands (TNF-*α* and FasL, resp.), produce a conformational change in the receptor and recruitment of RIP-1, TNFR- associated death domain (TRADD), and TNFR-associated factor (TRAF) 2 and 5 to the cell membrane. Together these components constitute complex I [32]. TRAF2 recruits the cellular inhibitor of apoptosis (cIAP) that allows polyubiquitylation of RIP-1 leading to activation of p38-mitogen-activated protein (MAP) kinase, nuclear factor- kappa B (NF*κ*B) and cell survival [37–40] (Figure 1). In a rodent model of neonatal HI, preservation of cIAP, via blockade of Smac/DIABLO, decreases injury size and improve outcomes [41], suggesting a possible role of RIP-1 ubiquitylation in cellular survival in this model. Likewise, preservation of RIP-1 ubiquitylation by genetic deletion of cylindromatosis (CYLD, deubiquitinating enzyme) in cultured cells results in resistance to TNF-induced programmed necrosis [42, 43] which persists despite zVAD-fmk treatment (pan-caspase inhibitor) [44]. The roles of caspase 8 (known to cleave CYLD [44]), CYLD, and ubiquitylation of RIP-1 in determining activation of signaling pathways for programmed necrosis or survival are entirely unexplored territory in the investigation of neonatal brain injury following HI. Furthermore, RIP-1 ubiquitylation and complex I have been recently linked to cell death via Nox1 activation suggesting that many other modulators may play an important role in the elaborate intracellular signaling leading to cell survival or death [45] (Figure 1).

In the setting of energy insufficiency, activation of TNFR signals for cellular death via a variety of mechanisms is triggered by the degree of energy deficit. If cellular energy is only partially limited, RIP-1 polyubiquitylation declines favoring the transition of complex I to cytosolic complex II via internalization of activated TNFR and formation of the death-inducing signaling complex (DISC) containing TRADD, Fas-associated protein (FADD) and procaspase 8 [32, 70, 71]. When RIP-1 kinase is active, caspase-8 is cleaved and activated, initiating the intrinsic and extrinsic apoptotic cascades [72]. Activated caspase 8 can then cleave RIP-1 and RIP-3 and consequently limit programmed necrosis [73, 74] (Figure 1). However, in the setting of more severe ATP depletion, caspase activity is inhibited allowing the formation of the RIP-1/RIP-3 complex, the necrosome, and cell death proceeds via programmed necrosis [10, 11, 75]. Interaction between RIP-1 and RIP-3 occurs at the RIP homotypic interaction motif (RHIM) which is the site of mutual phosphorylation [76]. Other RIP-1-dependent pathways do not require kinase activity as suggested by the lack of modulation of NF*κ*B following RIP-1 kinase blockade with necrostatin in cell culture [19]. Once again, no studies have addressed the formation of complex II *in vivo* following neonatal HI.

The interaction between FADD, RIP-1, and RIP-3 appears to be critical following TNFR activation [77]. RIP-1 is recruited to FADD in a TNF-dependent manner, while RIP-3 is more constitutively associated with FADD [78]. Following TNF exposure of cell cultures, FADD-deficient cells undergo RIP-3- and CYLD-dependent programmed necrosis with prominent inflammation, suggesting that FADD may prevent formation of the necrosome [79]. In addition to FADD, caspase 8 also seems to be necessary for survival of cultured cells due to its role in modulating CYLD activity and perhaps other functions [80]. In the developing mouse brain, there is abundant expression of caspase 8, TNFR, FAS death receptor, FADD, RIP-1, and RIP-3 [6, 7, 63]. In the normal developing brain, RIP-3 and FADD coimmunoprecipitate; following HI, RIP-1 is recruited to complex with RIP-3 disrupting RIP3's association with FADD [7]. These events are RIP-1 kinase dependent as proven by the partial restoration of RIP-3 and FADD association following treatment with necrostatin [7].

In the neonatal HI model, necrostatin not only provides neuroprotection but also partially shifts the death phenotype from necrosis to apoptosis validating the reality of the cell death continuum and providing insights into mechanisms that may drive the cell death continuum [6, 7]. A similar finding has been reported in cell culture; knockdown of RIP-1 prior to TNF*α* exposure switches cell death from necroptosis to apoptosis [42]. Some factors that may permit a switch from necrosis to apoptosis in mice treated with necrostatin early after HI are (i) preservation of the mitochondrial function and consequently ATP production, (ii) inhibition of FLIP ((Fas-associated death-domain-like IL-1*β* converting enzyme)-inhibitory protein) gene and protein expression [7, 81]; (iii) the fact that RIP-1 pathways leading to survival and apoptotic cell death are not kinase dependent [10, 19, 82]. We suspect that necrostatin-1, by blocking programmed necrosis, may allow a “cleaner” and less inflammatory form of cell death, similar to what is described for therapeutic hypothermia [83]. This possibility has not yet been explored.

### 2.2. Energy: The Driving Force

Mitochondrial dysfunction and energy failure is a hallmark in necrotic cell death following neonatal HI [6, 84–88]. RIP-1-dependent necroptosis evolves with increased reactive oxygen species (ROS) production, decreased ATP production, and decreased mitochondrial membrane potential [89]. In cultured cells, nitric oxide inhibits NADH dehydrogenase (mitochondrial complex I) causing depletion of intracellular ATP and promoting a switch from apoptosis to necrosis [33, 90, 91]. Nitric-oxide-(NO-) induced inhibition of mitochondrial complex I is reversible at low concentrations [91–93] but irreversible at high concentrations resulting in additional free radical production [94, 95]. After neonatal HI, inducible nitric oxide synthase (iNOS) expression and NO accumulation increase, events that are followed by a progressive decline in complex I activity in forebrain during the first 24 h (unpublished data, Pediatric Academic Society Meeting 2011 abstract 2170.2; Neuroscience 2012, submitted). This decline in complex I activity results in a significant impairment in ATP production at early stages following HI that is also prevented by blockade of RIP-1 kinase [96]. Blockade of RIP-1/RIP-3 complex formation in cell culture using necrostatin or RIP-1 siRNA prevents 3-nitrotyrosine accumulation and nitrosylation of complex I and attenuates NO-dependent necrosis [95] similar to findings in the neonatal *in vivo *HI model. These data are consistent with the hypothesis that an intact mitochondrion is initially required to produce physiological superoxide (O_2_^−^) that will react with NO to generate peroxynitrite (ONOO^−^) resulting in mitochondrial membrane potential loss [97, 98].

The link between programmed necrosis and opening of the mitochondrial permeability transition pore (MPTP) complex is controversial [22, 99]. However, RIP-1 appears to have direct effects in cellular energy production by translocating to the mitochondria and suppressing ADP/ATP exchange [20, 100] in cell culture. In concert with these findings, necrostatin also prevents the reduction in mitochondrial membrane potential caused by excitotoxic stimuli [101].

### 2.3. Free Radicals Targeting the Mitochondria

RIP-1 kinase activity is essential for cell death to proceed via the most well-recognized form of programmed necrosis. RIP-1 kinase activity mediates the formation of the necrosome (RIP-1/RIP-3 complex) which induces ROS production via effects on (i) Nox 1 nicotinamide adenine dinucleotide phosphate (NADPH) oxidase and (ii) the mitochondria [23, 45, 102]. Nevertheless, necrostatin is not a direct antioxidant and does not prevent cell death caused by hydrogen peroxide in culture [12, 103]. However, much like hypothermia, inhibition of RIP-1 kinase activity attenuates oxidative injury to proteins following neonatal HI in the mouse and piglet [7, 83]. Similarly, genetic deletion of RIP-3 gene or treatment with RIP-3 silencing RNA (siRNA) in cultured cells prevents increase in ROS and programmed cell necrosis [78]. Potential oxidative injury mechanisms targeted by the blockade of programmed necrosis include (i) blockade of nitric-oxide-mediated mitochondrial dysfunction caused by lipopolysaccharides (LPS) stimulation of macrophages [95], (ii) inhibition of glutamate excitotoxicity [103], (iii) increased glutathione levels [103], and (iv) decreased ROS production [103].

Glutathione (GSH) levels decrease following both excitotoxic and HI insults but blockade of RIP-1 kinase with necrostatin increases GSH production in HT-22 cells following glutamate exposure [45, 103]. In the neonatal HI mouse model, treatment with necrostatin appears to prevent glutathione oxidation rather than increasing GSH production *per se* [96]. This finding may reflect an indirect effect of the prevention of early protein carbonyl formation afforded by necrostatin-1 after neonatal HI [7] or it may simply be an indirect consequence of neural cell protection.

Recently, a role for Bcl-2/adenovirus E1B 19 kDa-interacting protein 3 (BNip3) has been described in a programmed necrotic-like cell death [104]. This BH3-only protein subfamily includes two members: BNip3 (also called NIP3) and BNip3L (also called NIX or BNip3-like) each with different recognized functions [105, 106]. BNip3 (30 kDa monomer) binds loosely to the outer mitochondria membrane (OMM) [107]. Free radical accumulation induces BNip3 dimerization and insertion into the OMM triggering necrotic-like cell death [104, 108]. In models of neonatal HI, necrostatin prevents early iNOS expression and NO accumulation and blocks hypoxia-inducible factor (HIF)-1*α* expression (unpublished data), a transcription factor that binds to the hypoxia response element (HRE) at the BNip3 promoter [109, 110]. Because NO modulates HIF-1*α* expression via Ras modification and phosphorylated extracellular-signal-regulated kinase (ERK) nuclear accumulation [109], it is possible that by preventing NO accumulation, necrostatin could indirectly decrease HIF-1*α* and consequently BNip3 expression following neonatal HI, protecting the mitochondria and preventing the progress of programmed necrosis. The second member of the BNip3 subfamily, BNip3L, has dual, but distinct, actions depending on the targeted organelle, mitochondria, or endoplasmic reticulum [106]. Although BNip3L has not been studied in models of neonatal HI, there is data from cellular cultures. At the mitochondria, BNip3L induces Bax/Bak-dependent OMM permeabilization, cytochrome c release, caspase activation and apoptosis, while, at the endoplasmic reticulum, BNip3L induces acute release of luminal Ca^2+^ that triggers cyclophilin-D-dependent MPTP complex opening, mitochondria swelling, mitochondrial membrane potential loss, ATP depletion, release of free radicals, and cellular necrosis [106]. Conversely, Bax/Bak has been also associated with programmed necrosis via release of AIF and mitochondrial depolarization [89, 111]. Therefore, both members of the BNip3 subfamily can be classified as sensors of mitochondrial stress as suggested previously [112] and because its expression is modulated by stimuli that are very well-recognized in association with HI, it is possible that both, BNip3 and BNip3L, are linked with the mitochondrial dysfunction seen following HI.

The pathways linking RIP-1 activity and RNS production are mostly unknown. Increased NO accumulation and iNOS expression potentiates glutamate release, *N*-methyl *D*-aspartate receptor (NMDAR) activity, necrotic neuronal death, and progression of excitotoxic injury in cell cultures [33, 113, 114]. Allosteric inhibition of RIP-1 kinase prevents the RNS formation as evidence by the decreased nitration of the NDUFB8 subunit preventing mitochondrial complex I dysfunction and depolarization [95]. Unpublished experiments from our laboratory are in agreement with these finding suggesting that blockade of RIP-1 kinase activity following neonatal HI decreases NO accumulation by 70% coincidently with a decrease in iNOS expression (unpublished data, Pediatric Academic Society Meeting 2011 abstract 2170.2). It remains unknown which mechanisms are operative and if they are directly linked to the inhibition of programmed necrosis. Anti-iNOS/NO effects of necrostatin may involve modulation of inflammatory mediators since cytokines are primary activators of iNOS production by astrocytes and necrostatin decreases cytokine expression [7, 115].

Ultimately, overproduced ROS and RNS attack the mitochondria, depleting ATP production and allowing programmed necrosis to proceed. ROS induces DNA alkylation, an event that increases the levels of calpain-dependent PARP-1 required for DNA repair [27, 28] in the setting of caspase 8 inhibition. Hyperactivity of PARP-1 following glutamate excitotoxicity produces poly-ADP-ribose (PAR) accumulation and ATP depletion inducing translocation of AIF from the mitochondria to the nucleus via a c-Jun-N-terminal-kinase-(JNK)-1-mediated mechanism resulting in chromatin condensation and DNA fragmentation [29, 30]. The importance of PARP-1 activation and AIF translocation in the neonatal brain after HI appears to be gender specific [26, 50]. PARP-1 level peaks at 30 min and again at 12 h following neonatal HI [68] along with an early decrease in nicotinamide adenine dinucleotide (NAD^+^) in male mice [26]. Furthermore, PARP-1 genetic deletion [26] or inhibition [67] provides neuroprotection following neonatal HI in male but not female mice. Blockade of calpains, required for PARP-1 activation, using MDL28170 [53] or hypothermia [54] or blocking JNK pathway [41] also decreases necrotic injury after HI. The degree of AIF translocation to the nucleus, also greater in male mice [50], correlates with the infarct size following neonatal HI [46] and its inhibition by heat shock protein (Hsp)-70 [48] TAT-Bcl-xL [49] or hypothermia [51] provides neuroprotection. Although still unclear, steps following PARP-1 activation may include RIP-1 activation as evidenced by the protection against DNA alkylation in RIP-1 knockdown mouse embryonic fibroblast [29]. Altogether, these data suggest an important role of a PARP-1-AIF feedback cycle in the events leading to brain injury following neonatal HI, direct evidence of interaction of AIF with RIP-1 (or the necrosome) has yet to be reported in the immature brain.

### 2.4. Inflammation and Programmed Necrosis

The importance of inflammation following HI has been extensively studied in the immature brain [116–118]. In normal physiology, a primary function of RIP-1 is to transduce the NF*κ*B signal leading to survival, hence RIP-deficient mice fail to thrive and die within three days after birth with extensive lymphoid apoptosis associated with failure to activate NF*κ*B due to unfavorable conditions to form complex I [32, 119]. Cell culture studies failed to show that RIP-1 kinase modulates NF*κ*B activation [19]. However, *in vivo*, we have shown that blockade of RIP-1 kinase activity using necrostatin following neonatal HI is associated with prevention of early increase in nuclear translocation of NF*κ*B [7]. This effect is likely indirect but may be of significance given the toxicity associated with early increases in NF*κ*B levels after neonatal HI [69, 120]. Additional confirmation of a possible indirect modulatory effect on NF*κ*B is that transcription of FLIP is downregulated following RIP-1 kinase blockade [7]. Because FLIP is under transcriptional control by NF*κ*B, the decline in early FLIP [121] expression following blockade of RIP-1 kinase with necrostatin may be a reporter for changes in NF*κ*B activity. 

NF*κ*B is a transcription factor that also mediates important apoptotic and inflammatory pathways which are central to HI-mediated brain injury in the immature brain [69, 120, 122]. Innate immune responses are dependent on activation of toll-like receptors (TLRs), recruitment of myeloid differentiation primary response gene (MyD)88 and interleukin-1 receptor-associated kinase (IRAK), association of TRAF6 and MAP3K, phosphorylation of I kappa B kinase (IKK) and release and nuclear translocation of the transcriptional factor NF*κ*B (p65/RelA/p50), resulting in change in cytokine expression [122]. Other proinflammatory receptors linked to NF*κ*B include the nucleotide-binding oligomerization domain (NOD) which with the interleukin (IL)-1 converting enzyme protease-activation factor (IPAF) activates caspase 1 (IL-1*β* converting enzyme) and forms the inflammasome [123–125]. Further details about the inflammatory pathways triggered by NF*κ*B activation may be reviewed elsewhere [122]. Current understanding of the “crosstalk” between programmed necrosis and inflammatory pathways is very limited; however certain interactions can be suspected based on current data. Blockade of programmed cell necrosis and cytokine expression in the neonatal HI model following treatment with necrostatin suggest that inhibition of RIP-1 kinase decreases the activation of the inflammasome, as shown by decreased caspase 1 activity and decreased transcription of IL-1*β* [7]. Furthermore, TNF-*α* and IL-6 are also downregulated in mice treated by necrostatin following neonatal HI, suggesting that RIP-1 kinase modulates neuroinflammation. However, it remains unclear if these anti-inflammatory changes are a direct effect of blockade of programmed necrosis pathway or whether they are secondary to the overall neuroprotection.

Although astrocytes provide support to neurons, they also release cytokines that instigate and perpetuate neuroinflammation [126]. TLR are constitutively expressed in astrocytes and modulation of these receptors following HI has been characterized [127]. Following induction of programmed necrosis, reactive astrocytes release cytokines and express iNOS [128], suggesting that changes in the cytokine profile associated with RIP-1 kinase blockade in HI may be related to an effect on astrocytes. Our preliminary results show that following neonatal HI, necrostatin decreases iNOS and cytokine expression while preserving astrocyte mitochondrial ultrastructure and attenuating glial fibrillary acidic protein (GFAP) expression at later stages. One possible hypothesis explaining the neuroprotective and anti-inflammatory effect associated with RIP-1 kinase inhibition is that *in vivo* astrocytes are a primary therapeutic target of necrostatin and by protecting and preserving astrocyte structure and function, it protects neurons and prevents neuroinflammation.

### 2.5. Gender Differences in Programmed Necrosis

Gender differences have been reported in neonatal rodent models of HI brain injury [7, 26, 50]. These differences may result from intrinsic differences in primary injury pathways. We found a more robust neuroprotection in males than females in response to programmed necrosis blockade [7]. Mechanisms explaining these gender differences are unresolved, but may involve an effect of necrostatin on the more significant decline in NAD^+^ following PARP-1 activation [26] and the preferential nuclear translocation of AIF [50] found in male rodents following neonatal HI. Therefore, necrostatin's blockade of RIP-1/RIP-3 interaction, oxidative damage, and inflammation may reflect mechanisms of action upstream and downstream of AIF translocation in male rodents.

## 3. Conclusions

Neonatal HI brain injury remains a common cause of developmental disability despite ongoing advances in obstetrical and neonatal care. With the advent of hypothermia for treatment of some infants with HI, morbidity has begun to decrease [129]. However, hypothermia is only partially neuroprotective after neonatal HI and 45% of all treated infants still suffer severe neurodevelopmental disability or death despite treatment [130]. Development of adjuvant therapies for hypothermia treatment has been limited to date. Novel approaches to understanding neurodegeneration after neonatal HI are needed. The conceptualization of the apoptosis-necrosis “continuum” in neonatal brain injury in 1997 predicted important mechanistic interactions between apoptosis and necrosis pathways [131]. Evidence of programmed necrosis in neonatal HI is in complete agreement with this sentinel observation and provides an important new direction for future research [7]. Programmed necrosis has been well studied in cellular cultures with new findings published routinely but the recognition of its importance in neonatal HI is just beginning. Many components of the signaling pathway now known to also regulate programmed necrosis have been studied over the last decade in models of neonatal HI as part of the apoptotic pathways showing the clear overlap of these pathways (Table 1). As we now begin to understand the contribution of programmed necrosis to neural cell fate following HI injury, we should take a fresh look at previous findings from these earlier studies. However, many questions remain unanswered with respect to programmed necrosis and neonatal HI including (i) direct effect, if any, of RIP-1 (or the necrosome) in disruption of mitochondrial bioenergetics; (ii) role of calpain-mediated lysosomal destabilization in the progression of injury; (iii) link between RIP-1 and PARP-1-AIF feedback cycle; (iv) identification of neural cell types most vulnerable programmed necrosis and the role of individual neural cell types in propagation or resistance to programmed necrosis; (v) the cellular mechanisms activated following necrosome formation in the immature brain; (vi) whether specific inhibitors of programmed necrosis will be clinically useful; (vii) what effect, if any, current therapies have on programmed necrosis following HI. Studies such as these will provide new perspectives on the mechanisms of neuronal cell death *in vivo* and may lay the foundation for new effective therapies for neonatal HI.

## Figures and Tables

**Table 1:** Components of continuum-programmed necrosis pathway in neonatal HI models.

Component	Finding	(Year) Researchers
AIF	Translocation from mitochondria to nucleus produces DNA condensation. ↑ is correlated with ↑ infarct size (Rat model)	(2003) Zhu et al. [46]
	
	AIF effect on DNA is nitric oxide independent (Rat Model)	(2004) Zhu et al. [47]
	
	Hsp-70 ↓ translocation of AIF to the nucleus (Mouse model)	(2005) Matsumori et al. [48]
	
	TAT-Bcl-xL ↓ AIF translocation to nucleus and caspase activation providing neuroprotection post HI (Rat model)	(2006) Yin et al. [49]
	
	↑ nuclear translocation in males associated with ↑ injury Female mice show greater caspase 3 activity. (Mouse model)	(2006) Zhu et al. [50]
	
	Hypothermia ↓ AIF translocation. (Rat model)	(2011) Askalan et al. [51]

Calpains	m-calpain but not *μ*-calpain cleaves caspase-3 (Rat model)	(2001) Blomgren et al. [52]
	
	Calpain inhibition (using MDL28170) provides neuroprotection and ↓ necrosis (Rat model)	(2005) Kawamura et al. [53]
	Prolonged hypothermia ↓ calpain activation (Rat Model)	(2005) Ohmura et al. [54]
	
	Polyphenols (pomegranate) provide neuroprotection and decrease calpain activation (Mouse model)	(2007) West et al. [55]
	
	Inhibition produced by inhibition of JNK (using D-JNKI1) (Rat model)	(2009) Ginet et al. [56]
	
	TAT-mGluR1 blocks the calpain cleavage site of mGluR1*α* and provide neuroprotection (Rat model)	(2009) Zhou et al. [57]
	Inhibition of JNK (using TAT-JBD) prevents calpain-mediated brain injury after HI (Rat model)	(2010) Nijboer et al. [41]
	
	Calpain modulates the ↓ in Bcl-2 following HI (Rat model)	(2010) Zhu et al. [58]
	
	Ethyl pyruvate is neuroprotective via inhibition of calpain activation and Ca^2+^ dysregulation. (Rat model)	(2010) Shen et al. [59]

Cathepsins	Propidium ioidide + cells in cortex and hippocampus were + for cathepsin B after HI suggesting necrosis (Rat model)	(2007) Carloni et al. [60]
	
	Cathepsin D ↑ at 6 h and 24 h post-HI (Rat model)	(2009) Ginet et al. [56]

FADD	Expression is independent of gluthatione levels and hydrogen peroxide accumulation (Mouse model)	(2007) Payton et al. [61]
	
	Inhibition of RIP-1 kinase activity restores the RIP-3/FADD interaction (Mouse model)	(2011) Northington et al. [7]

Fas-DR	↑ in the thalamus following HI along with ↑ cleavage of caspase 8. (Rat model)	(2001) Northington et al. [62]
	
	↑ after HI and genetic deletion provides neuroprotection to cortex (Mouse model)	(2004) Graham et al. [63]

Hsp-90	—	No *in vivo* HI studies

Hsp-70	Hsp-70 overexpression provide protection against apoptosis (Mouse model)	(2005) Matsumori et al. [48]
	
	↑ FLIP levels, ↓ caspase-8 and 9 cleavage, and cytochrome C translocation to cytosol (Mouse model)	(2006) Matsumori et al. [64]

JNK	Activated after HI. Genetic deletion ↓ brain tissue loss. Activates c-JUN, ATF-2, Bim/PUMA (Mouse model)	(2007) Pirianov et al. [65]
	
	Inhibition (using D-JNKI1), ↓ caspase-3 activation. (Rat model)	(2009) Ginet et al. [56]
	
	Inhibition (using TAT-JBD) ↓ injury, improves outcomes, and preserves IAP (via inhibition of Smac/DIABLO). (Rat model)	(2010) Nijboer et al. [41]

p53	↑ in mitochondria→↑ cytochrome C and Smac/DIABLO translocation. ↓ p53 →↓ infarct (better outcomes). (Rat model)	(2011) Nijboer et al. [66]

PARP-1	Activation after HI but ↓ NAD^+^ only in male mice and genetic deletion affords neuroprotection in males. (Mouse model)	(2004) Hagberg et al. [26]
	
	Simvastatin ↓ PARP-1 activation and IL-1*β* expression and provides neuroprotection (Rat model)	(2006) Carloni et al. [67]
	
	Immunoreactivity (IHC) peaks at 30 min and then again at 12 h post HI (Rat model)	(2005) Martin et al. [68]

RIP1/RIP3	↓ complex (necrosome) formation by necrostatin after HI affords neuroprotection, ↓ oxidation and FLIP (Mouse model)	(2011) Northington, et al. [7]

TNFR	NF-*κ*B inhibition ↓ brain damage and switches the HI-induced TNF-R profile from ↑ TNF-R1 to ↑ TNF-R2. (Rat model)	(2009) Nijboer et al. [69]

TRADD	—	No *in vivo* HI studies

AIF: apoptosis inducing factor; FADD: Fas-associated protein; Fas-DR: Fas death receptor; FLIP: (Fas-associated death-domain-like IL-1*β* converting enzyme)-inhibitory protein; HI: Hypoxia-ischemia, Hsp: heat shock protein; IAP: inhibitor of apoptosis JNK, Jun N-terminal kinase; NF*κ*B: nuclear factor-kappa B; PARP-1: Poly [ADP-ribose] polymerase-1; RIP: receptor interacting protein; TNFR: tumor necrosis factor receptor; TRADD: TNFR-associated death domain.

## Abbreviations

ATP: Adenosine-5′-triphosphate
AIF: Apoptosis inducing factor
BNIP: BCL2/adenovirus E1B 19 kDa protein-interacting protein
ASC: Caspase recruitment domain
cIAP: Cellular inhibitor of apoptosis
CYLD: Cylindromatosis
DISC: Death-inducing signaling complex
DNA: Deoxyribonucleic acid
ERK: Extracellular-signal-regulated kinase
FADD: Fas-associated protein
FLIP: (Fas-associated death-domain-like IL-1*β* converting enzyme)-inhibitory protein
Fas-DR: Fas death receptor
GFAP: Glial fibrillary acidic protein
GSH: Glutathione
Hsp: Heat shock protein
HIF: Hypoxia-inducible factor
HI: Hypoxia-ischemia;
HIE: Hypoxic-ischemic encephalopathy
HRE: Hypoxia response element
IKK: I kappa B kinase
iNOS: Inducible nitric oxide synthase
IL: Interleukin
IPAF: Interleukin (IL)-1 converting enzyme protease-activation factor
IRAK: Interleukin-1 receptor-associated kinase
JNK: Jun N-terminal kinase
LPS: Lipopolysaccharides
MyD: Myeloid differentiation primary response gene
MPTP: Mitochondrial permeability transition pore
MAP: Mitogen activated protein
NAD^+^: Nicotinamide adenine dinucleotide
NADPH: Nicotinamide adenine dinucleotide phosphate
NO: Nitric oxide
NMDAR: *N*-methyl *D*-aspartate receptor
NF*κ*B: Nuclear factor-kappa B
NOD: Nucleotide-binding oligomerization domain
OMM: Outer mitochondria membrane
PARP-1: Poly (ADP-ribose) polymerase 1
RNS: Reactive nitrogen species
RIP: Receptor interacting protein
RHIM: RIP homotypic interaction motif
ONOO^−^: Peroxynitrite
ROS: Reactive oxygen species
siRNA: Silencing ribonucleic acid
O_2_^−^: Superoxide
TLR: Toll-like receptors
TNF: Tumor necrosis factor
TNFR: Tumor necrosis factor receptor
TRADD: TNFR-associated death domain
TRAF: TNFR-associated factor.

## References

[1] Derrick M, Drobyshevsky A, Ji X, Tan S (2007). A model of cerebral palsy from fetal hypoxia-ischemia. *Stroke*.

[2] Myers WG (1975). The first radioindicator study in the life sciences with a man-made radionuclide: "Radioactive indicators in the study of phosphorus metabolism in rats, by O. Chievitz and G. Hevesy, reprinted from Nature 136: 754-755, Nov. 9, 1935. *Journal of Nuclear Medicine*.

[3] Rice JE, Vannucci RC, Brierley JB (1981). The influence of immaturity on hypoxic-ischemic brain damage in the rat. *Annals of Neurology*.

[4] Northington FJ, Ferriero DM, Martin LJ (2001). Neurodegeneration in the thalamus following neonatal hypoxia-ischemia is programmed cell death. *Developmental Neuroscience*.

[5] Towfighi J, Zec N, Yager J, Housman C, Vannucci RC (1995). Temporal evolution of neuropathologic changes in an immature rat model of cerebral hypoxia: a light microscopic study. *Acta Neuropathologica*.

[6] Northington FJ, Zelaya ME, O’Riordan DP (2007). Failure to complete apoptosis following neonatal hypoxia-ischemia manifests as "continuum" phenotype of cell death and occurs with multiple manifestations of mitochondrial dysfunction in rodent forebrain. *Neuroscience*.

[7] Northington FJ, Chavez-Valdez R, Graham EM, Razdan S, Gauda EB, Martin LJ (2011). Necrostatin decreases oxidative damage, inflammation, and injury after neonatal HI. *Journal of Cerebral Blood Flow and Metabolism*.

[8] Northington FJ, Chavez-Valdez R, Martin LJ (2011). Neuronal cell death in neonatal hypoxia-ischemia. *Annals of Neurology*.

[9] Chan FKM, Shisler J, Bixby JG (2003). A role for tumor necrosis factor receptor-2 and receptor-interacting protein in programmed necrosis and antiviral responses. *Journal of Biological Chemistry*.

[10] Holler N, Zaru R, Micheau O (2000). Fas triggers an alternative, caspase-8-independent cell death pathway using the kinase RIP as effector molecule. *Nature Immunology*.

[11] Vercammen D, Beyaert R, Denecker G (1998). Inhibition of caspases increases the sensitivity of L929 cells to necrosis mediated by tumor necrosis factor. *Journal of Experimental Medicine*.

[12] Berghe TV, Vanlangenakker N, Parthoens E (2010). Necroptosis, necrosis and secondary necrosis converge on similar cellular disintegration features. *Cell Death and Differentiation*.

[13] Dunai Z, Bauer PI, Mihalik R (2011). Necroptosis: biochemical, physiological and pathological aspects. *Pathology and Oncology Research*.

[14] Kung G, Konstantinidis K, Kitsis RN (2011). Programmed necrosis, not apoptosis, in the heart. *Circulation Research*.

[15] Peter ME (2011). Programmed cell death: apoptosis meets necrosis. *Nature*.

[16] Vandenabeele P, Galluzzi L, Vanden Berghe T, Kroemer G (2010). Molecular mechanisms of necroptosis: an ordered cellular explosion. *Nature Reviews Molecular Cell Biology*.

[17] Vanlangenakker N, Vanden Berghe T, Vandenabeele P (2012). Many stimuli pull the necrotic trigger, an overview. *Cell Death and Differentiation*.

[18] You Z, Savitz SI, Yang J (2008). Necrostatin-1 reduces histopathology and improves functional outcome after controlled cortical impact in mice. *Journal of Cerebral Blood Flow and Metabolism*.

[19] Degterev A, Hitomi J, Germscheid M (2008). Identification of RIP1 kinase as a specific cellular target of necrostatins. *Nature Chemical Biology*.

[20] Temkin V, Huang Q, Liu H, Osada H, Pope RM (2006). Inhibition of ADP/ATP exchange in receptor-interacting protein-mediated necrosis. *Molecular and Cellular Biology*.

[21] Degterev A, Huang Z, Boyce M (2005). Chemical inhibitor of nonapoptotic cell death with therapeutic potential for ischemic brain injury. *Nature Chemical Biology*.

[22] Lim SY, Davidson SM, Mocanu MM, Yellon DM, Smith CCT (2007). The cardioprotective effect of necrostatin requires the cyclophilin-D component of the mitochondrial permeability transition pore. *Cardiovascular Drugs and Therapy*.

[23] Shen HM, Pervaiz S (2006). TNF receptor superfamily-induced cell death: redox-dependent execution. *The FASEB Journal*.

[24] Motani K, Kushiyama H, Imamura R, Kinoshita T, Nishiuchi T, Suda T (2011). Caspase-1 protein induces apoptosis-associated speck-like protein containing a caspase recruitment domain (ASC)-mediated necrosis independently of its catalytic activity. *Journal of Biological Chemistry*.

[25] Tu HC, Ren D, Wang GX (2009). The p53-cathepsin axis cooperates with ROS to activate programmed necrotic death upon DNA damage. *Proceedings of the National Academy of Sciences of the United States of America*.

[26] Hagberg H, Wilson MA, Matsushita H (2004). PARP-1 gene disruption in mice preferentially protects males from perinatal brain injury. *Journal of Neurochemistry*.

[27] Los M, Mozoluk M, Ferrari D (2002). Activation and caspase-mediated inhibition of PARP: a molecular switch between fibroblast necrosis and apoptosis in death receptor signaling. *Molecular Biology of the Cell*.

[28] Moubarak RS, Yuste VJ, Artus C (2007). Sequential activation of poly(ADP-ribose) polymerase 1, calpains, and bax is essential in apoptosis-inducing factor-mediated programmed necrosis. *Molecular and Cellular Biology*.

[29] Xu Y, Huang S, Liu ZG, Han J (2006). Poly(ADP-ribose) polymerase-1 signaling to mitochondria in necrotic cell death requires RIP1/TRAF2-mediated JNK1 activation. *Journal of Biological Chemistry*.

[30] Yu SW, Wang H, Poitras MF (2002). Mediation of poty(ADP-ribose) polymerase-1—dependent cell death by apoptosis-inducing factor. *Science*.

[31] Eguchi Y, Shimizu S, Tsujimoto Y (1997). Intracellular ATP levels determine cell death fate by apoptosis or necrosis. *Cancer Research*.

[32] Micheau O, Tschopp J (2003). Induction of TNF receptor I-mediated apoptosis via two sequential signaling complexes. *Cell*.

[33] Leist M, Single B, Naumann H (1999). Inhibition of mitochondrial ATP generation by nitric oxide switches apoptosis to necrosis. *Experimental Cell Research*.

[34] Leist M, Single B, Castoldi AF, Kühnle S, Nicotera P (1997). Intracellular adenosine triphosphate (ATP) concentration: a switch in the decision between apoptosis and necrosis. *Journal of Experimental Medicine*.

[35] Leist M, Jäättelä M (2001). Four deaths and a funeral: from caspases to alternative mechanisms. *Nature Reviews Molecular Cell Biology*.

[36] Blomgren K, Leist M, Groc L (2007). Pathological apoptosis in the developing brain. *Apoptosis*.

[37] Ye Y-C, Yu L, Wang H-J, Tashiro S-I, Onodera S, Ikejima T (2011). TNF*α*-induced necroptosis and autophagy via supression of the p38-NF-*κ*B survival pathway in L929 cells. *Journal of Pharmacological Sciences*.

[38] Häcker H, Karin M (2006). Regulation and function of IKK and IKK-related kinases. *Science’s STKE*.

[39] Deveraux QL, Roy N, Stennicke HR (1998). IAPs block apoptotic events induced by caspase-8 and cytochrome c by direct inhibition of distinct caspases. *EMBO Journal*.

[40] Bertrand MJM, Milutinovic S, Dickson KM (2008). cIAP1 and cIAP2 facilitate cancer cell survival by functioning as E3 ligases that promote RIP1 ubiquitination. *Molecular Cell*.

[41] Nijboer CH, van der Kooij MA, van Bel F, Ohl F, Heijnen CJ, Kavelaars A (2010). Inhibition of the JNK/AP-1 pathway reduces neuronal death and improves behavioral outcome after neonatal hypoxic-ischemic brain injury. *Brain, Behavior, and Immunity*.

[42] Vanlangenakker N, Bertrand MJM, Bogaert P, Vandenabeele P, Vanden Berghe T (2011). TNF-induced necroptosis in L929 cells is tightly regulated by multiple TNFR1 complex i and II members. *Cell Death and Disease*.

[43] Hitomi J, Christofferson DE, Ng A (2008). Identification of a molecular signaling network that regulates a cellular necrotic cell death pathway. *Cell*.

[44] O'Donnell MA, Perez-Jimenez E, Oberst A (2011). Caspase 8 inhibits programmed necrosis by processing CYLD. *Nature Cell Biology*.

[45] Kim YS, Morgan MJ, Choksi S, Liu ZG (2007). TNF-induced activation of the Nox1 NADPH oxidase and its role in the induction of necrotic cell death. *Molecular Cell*.

[46] Zhu C, Qiu L, Wang X (2003). Involvement of apoptosis-inducing factor in neuronal death after hypoxia-ischemia in the neonatal rat brain. *Journal of Neurochemistry*.

[47] Zhu C, Wang X, Qiu L, Peeters-Scholte C, Hagberg H, Blomgren K (2004). Nitrosylation precedes caspase-3 activation and translocation of apoptosis-inducing factor in neonatal rat cerebral hypoxia-ischaemia. *Journal of Neurochemistry*.

[48] Matsumori Y, Hong SM, Aoyama K (2005). Hsp70 overexpression sequesters AIF and reduces neonatal hypoxic/ischemic brain injury. *Journal of Cerebral Blood Flow and Metabolism*.

[49] Yin W, Cao G, Johnnides MJ (2006). TAT-mediated delivery of Bcl-xL protein is neuroprotective against neonatal hypoxic-ischemic brain injury via inhibition of caspases and AIF. *Neurobiology of Disease*.

[50] Zhu C, Xu F, Wang X (2006). Different apoptotic mechanisms are activated in male and female brains after neonatal hypoxia-ischaemia. *Journal of Neurochemistry*.

[51] Askalan R, Wang C, Shi H, Armstrong E, Yager JY (2011). The effect of postischemic hypothermia on apoptotic cell death in the neonatal rat brain. *Developmental Neuroscience*.

[52] Blomgren K, Zhu C, Wang X (2001). Synergistic activation of caspase-3 by m-calpain after neonatal hypoxia-ischemia: a mechanism of "pathological apoptosis"?. *Journal of Biological Chemistry*.

[53] Kawamura M, Nakajima W, Ishida A, Ohmura A, Miura S, Takada G (2005). Calpain inhibitor MDL 28170 protects hypoxic-ischemic brain injury in neonatal rats by inhibition of both apoptosis and necrosis. *Brain Research*.

[54] Ohmura A, Nakajima W, Ishida A (2005). Prolonged hypothermia protects neonatal rat brain against hypoxic-ischemia by reducing both apoptosis and necrosis. *Brain and Development*.

[55] West T, Atzeva M, Holtzman DM (2007). Pomegranate polyphenols and resveratrol protect the neonatal brain against hypoxic-ischemic injury. *Developmental Neuroscience*.

[56] Ginet V, Puyal J, Magnin G, Clarke PGH, Truttmann AC (2009). Limited role of the c-Jun N-terminal kinase pathway in a neonatal rat model of cerebral hypoxia-ischemia. *Journal of Neurochemistry*.

[57] Zhou M, Xu W, Liao G, Bi X, Baudry M (2009). Neuroprotection against neonatal hypoxia/ischemia-induced cerebral cell death by prevention of calpain-mediated mGluR1*α* truncation. *Experimental Neurology*.

[58] Zhu C, Hallin U, Ozaki Y (2010). Nuclear translocation and calpain-dependent reduction of Bcl-2 after neonatal cerebral hypoxia-ischemia. *Brain, Behavior, and Immunity*.

[59] Shen H, Hu X, Liu C (2010). Ethyl pyruvate protects against hypoxic-ischemic brain injury via anti-cell death and anti-inflammatory mechanisms. *Neurobiology of Disease*.

[60] Carloni S, Carnevali A, Cimino M, Balduini W (2007). Extended role of necrotic cell death after hypoxia-ischemia-induced neurodegeneration in the neonatal rat. *Neurobiology of Disease*.

[61] Payton KSE, Sheldon RA, Mack DW (2007). Antioxidant status alters levels of fas-associated death domain-like IL-1B-converting enzyme inhibitory protein following neonatal hypoxia-ischemia. *Developmental Neuroscience*.

[62] Northington FJ, Ferriero DM, Flock DL, Martin LJ (2001). Delayed neurodegeneration in neonatal rat thalamus after hypoxia-ischemia is apoptosis. *Journal of Neuroscience*.

[63] Graham EM, Sheldon RA, Flock DL (2004). Neonatal mice lacking functional Fas death receptors are resistant to hypoxic-ischemic brain injury. *Neurobiology of Disease*.

[64] Matsumori Y, Northington FJ, Hong SM (2006). Reduction of caspase-8 and -9 cleavage is associated with increased c-FLIP and increased binding of Apaf-1 and Hsp70 after neonatal hypoxic/ischemic injury in mice overexpressing Hsp70. *Stroke*.

[65] Pirianov G, Brywe KG, Mallard C (2007). Deletion of the c-Jun N-terminal kinase 3 gene protects neonatal mice against cerebral hypoxic-ischaemic injury. *Journal of Cerebral Blood Flow and Metabolism*.

[66] Nijboer CH, Heijnen CJ, Van Der Kooij MA (2011). Targeting the p53 pathway to protect the neonatal ischemic brain. *Annals of Neurology*.

[67] Carloni S, Mazzoni E, Cimino M (2006). Simvastatin reduces caspase-3 activation and inflammatory markers induced by hypoxia-ischemia in the newborn rat. *Neurobiology of Disease*.

[68] Martin SS, Perez-Polo JR, Noppens KM, Grafe MR (2005). Biphasic changes in the levels of poly(ADP-ribose) polymerase-1 and caspase 3 in the immature brain following hypoxia-ischemia. *International Journal of Developmental Neuroscience*.

[69] Nijboer CH, Heijnen CJ, Groenendaal F, Van Bel F, Kavelaars A (2009). Alternate pathways preserve tumor necrosis factor-*α* production after nuclear factor-*κ*B inhibition in neonatal cerebral hypoxia-ischemia. *Stroke*.

[70] Christofferson DE, Yuan J (2010). Necroptosis as an alternative form of programmed cell death. *Current Opinion in Cell Biology*.

[71] Ea CK, Deng L, Xia ZP, Pineda G, Chen ZJ (2006). Activation of IKK by TNF*α* requires site-specific ubiquitination of RIP1 and polyubiquitin binding by NEMO. *Molecular Cell*.

[72] Vandenabeele P, Vanden Berghe T, Festjens N (2006). Caspase inhibitors promote alternative cell death pathways. *Science’s STKE*.

[73] Wang L, Du F, Wang X (2008). TNF-*α* induces two distinct caspase-8 activation pathways. *Cell*.

[74] Feng S, Yang Y, Mei Y (2007). Cleavage of RIP3 inactivates its caspase-independent apoptosis pathway by removal of kinase domain. *Cellular Signalling*.

[75] Declercq W, Vanden Berghe T, Vandenabeele P (2009). RIP kinases at the crossroads of cell death and survival. *Cell*.

[76] Sun X, Yin J, Starovasnik MA, Fairbrother WJ, Dixit VM (2002). Identification of a novel homotypic interaction motif required for the phosphorylation of receptor-interacting protein (RIP) by RIP3. *Journal of Biological Chemistry*.

[77] Thakar J, Schleinkofer K, Borner C, Dandekar T (2006). RIP death domain structural interactions implicated in TNF-mediated proliferation and survival. *Proteins*.

[78] Cho Y, Challa S, Moquin D (2009). Phosphorylation-driven assembly of the RIP1-RIP3 complex regulates programmed necrosis and virus-induced inflammation. *Cell*.

[79] Bonnet M, Preukschat D, Welz P-S (2011). The adaptor protein FADD protects epidermal keratinocytes from necroptosis in vivo and prevents skin inflammation. *Immunity*.

[80] Lu JV, Weist BM, Van Raam BJ (2011). Complementary roles of Fas-associated death domain (FADD) and receptor interacting protein kinase-3 (RIPK3) in T-cell homeostasis and antiviral immunity. *Proceedings of the National Academy of Sciences of the United States of America*.

[81] Kreuz S, Siegmund D, Rumpf JJ (2004). NF*κ*B activation by Fas is mediated through FADD, caspase-8, and RIP and is inhibited by FLIP. *Journal of Cell Biology*.

[82] Lee TH, Shank J, Cusson N, Kelliher MA (2004). The kinase activity of Rip1 is not required for tumor necrosis factor-*α*-induced I*κ*B kinase or p38 MAP kinase activation or for the ubiquitination of Rip1 by Traf2. *Journal of Biological Chemistry*.

[83] Mueller-Burke D, Koehler RC, Martin LJ (2008). Rapid NMDA receptor phosphorylation and oxidative stress precede striatal neurodegeneration after hypoxic ischemia in newborn piglets and are attenuated with hypothermia. *International Journal of Developmental Neuroscience*.

[84] Blomgren K, Hagberg H (2006). Free radicals, mitochondria, and hypoxia-ischemia in the developing brain. *Free Radical Biology and Medicine*.

[85] Fritz KI, Groenendaal F, Andersen C, Ohnishi ST, Mishra OP, Delivoria-Papadopoulos M (1999). Deleterious brain cell membrane effects after NMDA receptor antagonist administration to newborn piglets. *Brain Research*.

[86] Vannucci RC, Brucklacher RM, Vannucci SJ (1996). The effect of hyperglycemia on cerebral metabolism during hypoxia- ischemia in the immature rat. *Journal of Cerebral Blood Flow and Metabolism*.

[87] Vannucci RC, Towfighi J, Vannucci SJ (2004). Secondary energy failure after cerebral hypoxia-ischemia in the immature rat. *Journal of Cerebral Blood Flow and Metabolism*.

[88] Vannucci SJ, Seaman LB, Vannucci RC (1996). Effects of hypoxia-ischemia on GLUT1 and GLUT3 glucose transporters in immature rat brain. *Journal of Cerebral Blood Flow and Metabolism*.

[89] Irrinki KM, Mallilankaraman K, Thapa RJ (2011). Requirement of FADD, NEMO, and BAX/BAK for aberrant mitochondrial function in tumor necrosis factor alpha-induced necrosis. *Molecular and Cellular Biology*.

[90] Nicotera P, Leist M, Ferrando-May E (1998). Intracellular ATP, a switch in the decision between apoptosis and necrosis. *Toxicology Letters*.

[91] Riobó NA, Clementi E, Melani M (2001). Nitric oxide inhibits mitochondrial NADH:ubiquinone reductase activity through peroxynitrite formation. *Biochemical Journal*.

[92] Beltrán B, Mathur A, Duchen MR, Erusalimsky JD, Moncada S (2000). The effect of nitric oxide on cell respiration: a key to understanding its role in cell survival or death. *Proceedings of the National Academy of Sciences of the United States of America*.

[93] Chinta SJ, Andersen JK (2006). Reversible inhibition of mitochondrial complex I activity following chronic dopaminergic glutathione depletion in vitro: implications for Parkinson’s disease. *Free Radical Biology and Medicine*.

[94] Beckman JS, Koppenol WH (1996). Nitric oxide, superoxide, and peroxynitrite: the good, the bad, and the ugly. *American Journal of Physiology*.

[95] Davis CW, Hawkins BJ, Ramasamy S (2010). Nitration of the mitochondrial complex I subunit NDUFB8 elicits RIP1- and RIP3-mediated necrosis. *Free Radical Biology and Medicine*.

[96] Chavez-Valdez R, Martin LJ, Flock DL, Northington FJ RIP-1 Kinase Inhibition attenuates mitochondrial dysfunction in neurons and astrocytes following neonatal hypoxia-ischemia.

[97] Guidarelli A, Cerioni L, Cantoni O (2007). Inhibition of complex III promotes loss of Ca^2+^ dependence for mitochondrial superoxide formation and permeability transition evoked by peroxynitrite. *Journal of Cell Science*.

[98] Whiteman M, Armstrong JS, Cheung NS (2004). Peroxynitrite mediates calcium-dependent mitochondrial dysfunction and cell death via activation of calpains. *The FASEB Journal*.

[99] Smith CCT, Davidson SM, Lim SY, Simpkin JC, Hothersall JS, Yellon DM (2007). Necrostatin: a potentially novel cardioprotective agent?. *Cardiovascular Drugs and Therapy*.

[100] Wagener N, Ackermann M, Funes S, Neupert W (2011). A pathway of protein translocation in mitochondria mediated by the AAA-ATPase Bcs1. *Molecular Cell*.

[101] Hsu TS, Yang PM, Tsai JS, Lin LY (2009). Attenuation of cadmium-induced necrotic cell death by necrostatin-1: potential necrostatin-1 acting sites. *Toxicology and Applied Pharmacology*.

[102] Lin Y, Choksi S, Shen HM (2004). Tumor necrosis factor-induced nonapoptotic cell death requires receptor-interacting protein-mediated cellular reactive oxygen species accumulation. *Journal of Biological Chemistry*.

[103] Xu X, Chua CC, Kong J (2007). Necrostatin-1 protects against glutamate-induced glutathione depletion and caspase-independent cell death in HT-22 cells. *Journal of Neurochemistry*.

[104] Vande Velde C, Cizeau J, Dubik D (2000). BNIP3 and genetic control of necrosis-like cell death through the mitochondrial permeability transition pore. *Molecular and Cellular Biology*.

[105] Chen G, Cizeau J, Velde CV (1999). Nix and Nip3 form a subfamily of pro-apoptotic mitochondrial proteins. *Journal of Biological Chemistry*.

[106] Chen Y, Lewis W, Diwan A, Cheng EHY, Matkovich SJ, Dorn GW (2010). Dual autonomous mitochondrial cell death pathways are activated by Nix/BNip3L and induce cardiomyopathy. *Proceedings of the National Academy of Sciences of the United States of America*.

[107] Yook YH, Kang KH, Maeng O (2004). Nitric oxide induces BNIP3 expression that causes cell death in macrophages. *Biochemical and Biophysical Research Communications*.

[108] Kubli DA, Quinsay MN, Huang C, Lee Y, Gustafsson AB (2008). Bnip3 functions as a mitochondrial sensor of oxidative stress during myocardial ischemia and reperfusion. *American Journal of Physiology*.

[109] An HJ, Maeng O, Kang KH (2006). Activation of Ras up-regulates pro-apoptotic BNIP3 in nitric oxide-induced cell death. *Journal of Biological Chemistry*.

[110] Bruick RK (2000). Expression of the gene encoding the proapoptotic Nip3 protein is induced by hypoxia. *Proceedings of the National Academy of Sciences of the United States of America*.

[111] Cabon L, Galán-Malo P, Bouharrour A (2012). BID regulates AIF-mediated caspase-independent necroptosis by promoting BAX activation. *Cell Death and Differentiation*.

[112] Chinnadurai G, Vijayalingam S, Gibson SB (2008). BNIP3 subfamily BH3-only proteins: mitochondrial stress sensors in normal and pathological functions. *Oncogene*.

[113] Hewett SJ, Muir JK, Lobner D, Symons A, Choi DW (1996). Potentiation of oxygen-glucose deprivation-induced neuronal death after induction of iNOS. *Stroke*.

[114] Nicotera P, Lipton SA (1999). Excitotoxins in neuronal apoptosis and necrosis. *Journal of Cerebral Blood Flow and Metabolism*.

[115] Faraco G, Fossati S, Bianchi ME (2007). High mobility group box 1 protein is released by neural cells upon different stresses and worsens ischemic neurodegeneration in vitro and in vivo. *Journal of Neurochemistry*.

[116] Barks JDE, Liu YQ, Shangguan Y, Li J, Pfau J, Silverstein FS (2008). Impact of indolent inflammation on neonatal hypoxic-ischemic brain injury in mice. *International Journal of Developmental Neuroscience*.

[117] Pimentel VC, Pinheiro FV, De Bona KS (2011). Hypoxic-ischemic brain injury stimulates inflammatory response and enzymatic activities in the hippocampus of neonatal rats. *Brain Research*.

[118] Wixey JA, Reinebrant HE, Buller KM (2011). Inhibition of neuroinflammation prevents injury to the serotonergic network after hypoxia-ischemia in the immature rat brain. *Journal of Neuropathology and Experimental Neurology*.

[119] Kelliher MA, Grimm S, Ishida Y, Kuo F, Stanger BZ, Leder P (1998). The death domain kinase RIP mediates the TNF-induced NF-*κ*B signal. *Immunity*.

[120] Nijboer CH, Heijnen CJ, Groenendaal F, May MJ, Van Bel F, Kavelaars A (2008). A dual role of the nf-kappa b pathway in neonatal hypoxic-ischemic brain damage. *Stroke*.

[121] Okada Y, Kato M, Minakami H (2001). Reduced expression of flice-inhibitory protein (FLIP) and NF*κ*B is associated with death receptor-induced cell death in human aortic endothelial cells (HAECs). *Cytokine*.

[122] Kucharczak J, Simmons MJ, Fan Y, Gélinas C (2003). To be, or not to be: NF-*κ*B is the answer—role of Rel/NF-*κ*B in the regulation of apoptosis. *Oncogene*.

[123] Martinon F, Burns K, Tschopp J (2002). The Inflammasome: a molecular platform triggering activation of inflammatory caspases and processing of proIL-*β*. *Molecular Cell*.

[124] Poyet JL, Srinivasula SM, Tnani M, Razmara M, Fernandes-Alnemri T, Alnemri ES (2001). Identification of Ipaf, a human caspase-1-activating protein related to Apaf-1. *Journal of Biological Chemistry*.

[125] Yoo NJ, Park WS, Kim SY (2002). Nod1, a CARD protein, enhances pro-interleukin-1*β* processing through the interaction with pro-caspase-1. *Biochemical and Biophysical Research Communications*.

[126] Freeman MR (2010). Specification and morphogenesis of astrocytes. *Science*.

[127] Stridh L, Smith PLP, Naylor AS, Wang X, Mallard C (2011). Regulation of Toll-like receptor 1 and -2 in neonatal mice brains after hypoxia-ischemia. *Journal of Neuroinflammation*.

[128] Laird MD, Wakade C, Alleyne CH, Dhandapani KM (2008). Hemin-induced necroptosis involves glutathione depletion in mouse astrocytes. *Free Radical Biology and Medicine*.

[129] Shah PS, Ohlsson A, Perlman M (2007). Hypothermia to treat neonatal hypoxic ischemic encephalopathy: systematic review. *Archives of Pediatrics and Adolescent Medicine*.

[130] Shankaran S, Laptook AR, Ehrenkranz RA (2005). Whole-body hypothermia for neonates with hypoxic-ischemic encephalopathy. *New England Journal of Medicine*.

[131] Portera-Cailliau C, Price DL, Martin LJ (1997). Excitotoxic neuronal death in the immature brain is an apoptosis- necrosis morphological continuum. *Journal of Comparative Neurology*.

